# Frequency dispersion amplifies tsunamis caused by outer-rise normal faults

**DOI:** 10.1038/s41598-021-99536-x

**Published:** 2021-10-08

**Authors:** Toshitaka Baba, Naotaka Chikasada, Kentaro Imai, Yuichiro Tanioka, Shuichi Kodaira

**Affiliations:** 1 Graduate School of Technology, Industrial and Social Sciences, Tokushima University, 2-1 Minami-jyosanjima-cho, Tokushima, 770-8506 Japan; 2 National Research Institute for Earth Science and Disaster Resilience, Ibaraki, Japan; 3 Japan Agency for Marine-Earth Science and Technology, Yokohama, Japan; 4 Hokkaido University, Sapporo, Hokkaido Japan

**Keywords:** Natural hazards, Ocean sciences, Solid Earth sciences

## Abstract

Although tsunamis are dispersive water waves, hazard maps for earthquake-generated tsunamis neglect dispersive effects because the spatial dimensions of tsunamis are much greater than the water depth, and dispersive effects are generally small. Furthermore, calculations that include non-dispersive effects tend to predict higher tsunamis than ones that include dispersive effects. Although non-dispersive models may overestimate the tsunami height, this conservative approach is acceptable in disaster management, where the goal is to save lives and protect property. However, we demonstrate that offshore frequency dispersion amplifies tsunamis caused by outer-rise earthquakes, which displace the ocean bottom downward in a narrow area, generating a dispersive short-wavelength and pulling-dominant (water withdrawn) tsunami. We compared observational evidence and calculations of tsunami for a 1933 *M*_*w*_ 8.3 outer-rise earthquake along the Japan Trench. Dispersive (Boussinesq) calculations predicted significant frequency dispersion in the 1933 tsunami. The dispersive tsunami deformation offshore produced tsunami inundation heights that were about 10% larger than those predicted by non-dispersive (long-wave) calculations. The dispersive tsunami calculations simulated the observed tsunami inundation heights better than did the non-dispersive tsunami calculations. Contrary to conventional practice, we conclude that dispersive calculations are essential when preparing deterministic hazard maps for outer-rise tsunamis.

## Introduction

Sudden seafloor displacements by earthquakes and submarine landslides displace the overlying water column, generating tsunamis. The great 2004 Indian Ocean tsunami, following the Sumatra earthquake (*M*_*w*_ 9.1)^1–4^ that ruptured the plate boundary between the Indian, the Australian, and the Eurasian plates, caused massive damage across vast coastal regions around the Indian Ocean and run-ups as high as 50 m above sea level^5^. Although the Japan Meteorological Agency immediately issued a tsunami warning following the 2011 *M*_*w*_ 9.0 interplate earthquake in the Japan Trench subduction zone^6–9^, the ensuing tsunami killed about 20,000 people. Mori et al.^10^ measured a maximum tsunami run-up of 40.4 m at Aneyoshi in Iwate in northeastern Japan. Tsunami disasters of this kind are too frequent worldwide to list them all, but they include the 2010 Maule (Chile)^11^, the 2013 Solomon^12^, and the 2018 Palu tsunamis (Indonesia)^13^. Mitigating tsunami disasters is thus an enormous global issue.

Other large earthquakes generated tsunamis in the Japan Trench subduction zone in 1896 and 1933. The 1896 *M*_*s*_ 7.2 Meiji-Sanriku interplate earthquake^14^ caused a huge tsunami that was larger than that expected from the shaking intensity alone^15–17^. Because of the minimal shaking, residents were not aware of the tsunami danger. Shuto et al.^18^ estimated the number of fatalities to be about 22,000, or roughly equal to that of the 2011 Tohoku tsunami. In 1933, 39 years after the 1896 earthquake, the *M*_*w*_ 8.3 normal-faulting Showa-Sanriku earthquake occurred on an outer-rise fault in the subducting Pacific plate^19–21^. This earthquake might have been relevant to the 1896 earthquake because interplate earthquakes enhance bending in the subducting plate. Because the epicenter of the 1933 earthquake was far from land, seaward of the Japan Trench axis, the seismic intensity on land (5 on the JMA scale) was smaller than that observed during the 2011 Tohoku earthquake (7 on the JMA scale). The 1933 earthquake, however, produced a large tsunami that caused severe damage comparable that of the 2011 Tohoku tsunami. The maximum run-up of the 1933 tsunami reached 28.7 m and about 3,000 people died^18^. Another famous example of an interplate/outer-rise earthquake pair occurred in the Kuril Trench; the 2006 *M 8.3* Kuril interplate earthquake was followed by the 2007 *M* 8.1 Kuril outer-rise earthquake 2 months later^22–25^. The 1977 *M*_*w*_ 8.2 Sumba earthquake was one of the most powerful outer-rise normal-faulting events ever recorded^26, 27^. In 2012, a large intraplate earthquake (*M*_*w*_ 8.6) occurred in the subducting plate off the source region of the 2004 Sumatra earthquake^28–30^. Although the focal mechanism of that event indicates strike-slip fault motion, it may have changed from pure normal faulting because of the characteristic geological setting or the state of pre-accumulated stresses. Accordingly, intraplate earthquakes in a subducting plate can be activated by neighboring large interplate earthquakes. However, an outer‐rise earthquake corresponding to the 2011 Tohoku earthquake has yet to occur in the Japan Trench subduction zone. Although a *M*_*w*_ 7.6 outer-rise normal faulting earthquake occurred about 40 min after the 2011 main shock, its magnitude was much smaller than that of the main shock.

Here, we model possible future tsunamis that might be caused by a large outer-rise earthquake that could follow the 2011 Tohoku event. Tsunami hazard maps^31–35^ are the most basic element in mitigating tsunami disasters, showing tsunami risks such as expected inundation area, flow depth, and arrival time. Because hazard maps also indicate locations and accessible evacuation routes, they are useful for rapid evacuations during tsunami disasters. Authorities in many tsunami-prone regions have already prepared deterministic tsunami hazard maps and disseminated them to residents. The construction of a deterministic tsunami hazard map consists of defining a set of potential tsunami source models that incorporate the maximum tsunami size expected based on geophysical knowledge of a region or the maximum tsunami size the region has ever experienced. These tsunami source models are then used in numerical simulations. Because tsunami propagation depends on water depth, the accuracy of the bathymetric and topographic data control prediction accuracy. For tsunami propagation and inundation calculations, the Navier-Stokes equations, the universal governing equations of fluid flow, can be used to accurately predict seawater motions during tsunamis. But seawater can be treated as an incompressible and non-viscous fluid. Also, a tsunami can be approximated by a long-wave because the fault length for a great tsunami is several hundred kilometers, whereas the ocean depth is only about 5 km. Accordingly, the long-wave (shallow water) theory^36^ is adequately accurate for constructing deterministic tsunami hazard maps. The calculation requirements for solving the long-wave equations are small enough so that a personal computer can be used to solve the equations.

**Figure 1:**
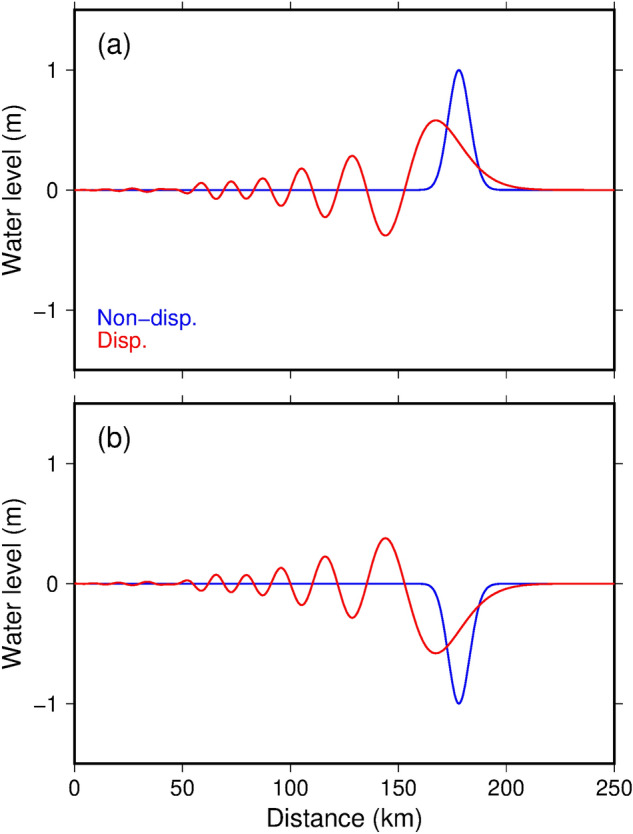
Non-dispersive (blue) and dispersive (red) propagation of Gaussian soliton wave shapes. (**a**) A positive (upwardly convex) incident wave. (**b**) A negative (downwardly convex) incident wave. The waves propagate in the horizontal direction over a water depth of 4000 m. Dispersion creates wave trains and increases the wave height in (**b**). Movie S1 shows propagations of the waves.

We followed this general procedure, but also incorporated dispersive (Boussinesq) terms into the long-wave equations^37, 38^ to improve the accuracy of the tsunami calculations. Because the phase velocity of a gravity wave in a dispersive model depends on water depth and wavelength, wave dispersion cannot be neglected for some classes of tsunami sources that have short wavelengths. In general, dispersion delays the short-wavelength component of tsunamis, forms wave trains, and reduces the maximum water height^39, 40^. High-angle normal faults in the subducting plate cause outer-rise earthquakes under the deep ocean. The earthquakes deform the ocean bottom by subsidence in a narrow area, resulting in the generation of a pulling-dominant (water withdrawn), short-wavelength tsunami. Therefore, dispersion in an outer-rise tsunami is not negligible, as has been observed by bottom pressure gauges in the deep ocean where nonlinear effects on tsunami propagation are small^41–43^. However, the effects of dispersion are often neglected in tsunami hazard maps because of the high computational costs associated with dispersive tsunami calculations. Compared to dispersive calculations, non-dispersive (long-wave) calculations also tend to overpredict tsunami heights in our estimations (Fig. 1a, and Movie S1a) and previous studies^39, 40^. Such conservative, non-dispersive approaches are generally acceptable in disaster management, where the goal is to save lives and protect property.

## Outer-rise fault models in the Japan Trench

**Figure 2:**
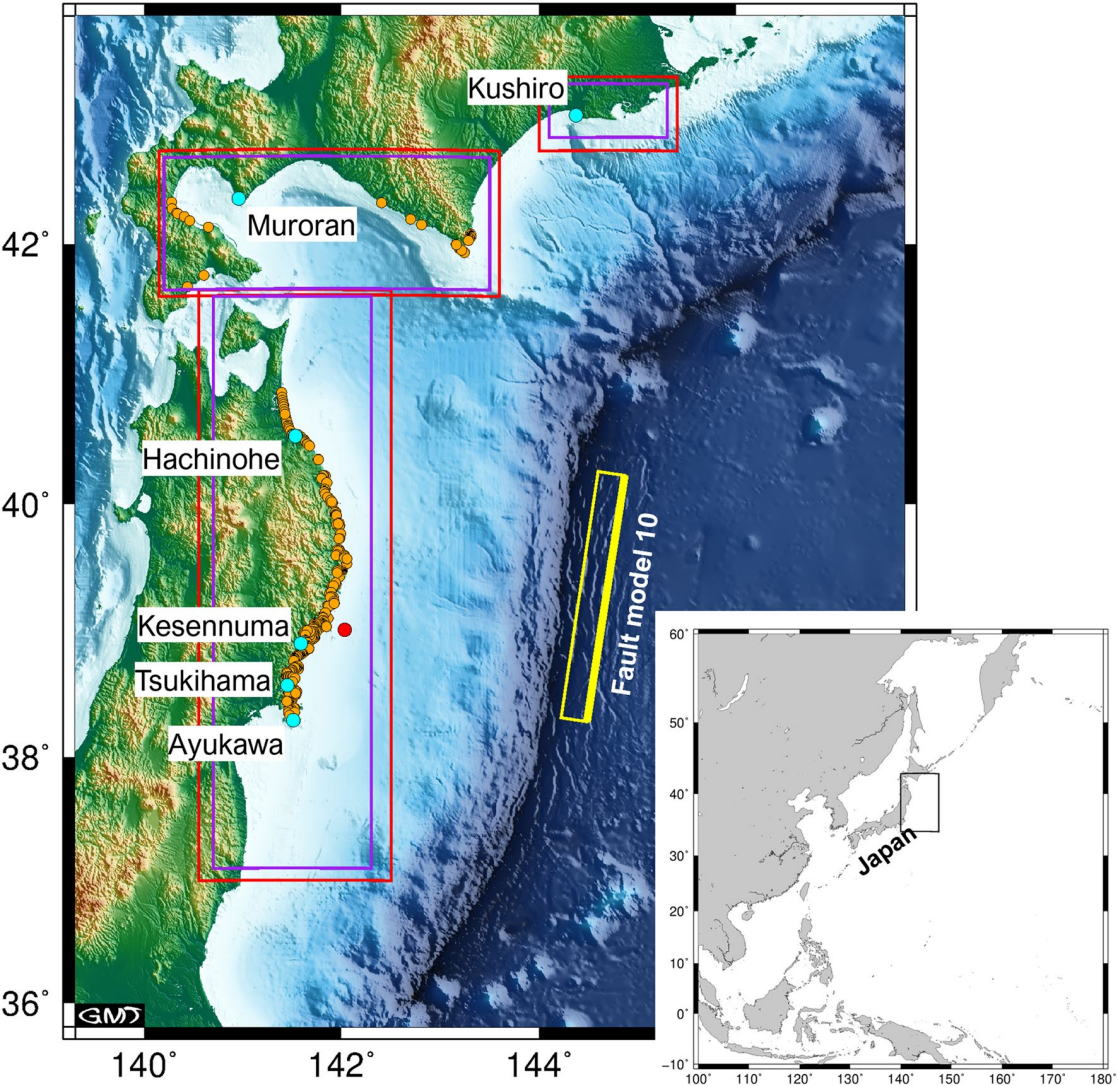
Map of the tsunami simulation region showing the distribution of the observed tsunami inundation data for the 1933 Showa-Sanriku tsunami (orange circles). The narrow yellow rectangle is a projection of the outer edge of fault model 10 (Table S1). The thick yellow line is the upper edge of the fault. Red and purple rectangles are the nested 6-arc-sec and 2-arc-sec gridded domains, respectively, used in the tsunami simulations. The root domain is gridded by 18-arc-sec intervals. The red circle indicates the location of the tsunami waveforms shown in Fig. 5. The cyan circles indicate the tide gauges used in Fig. 7. We used GMT^64^ Version 5.4.5 to prepare the map.

Our initial purpose was to accurately predict tsunamis caused by outer-rise earthquakes in the Japan Trench. Following the general procedure describe above, Baba et al.^41^ constructed outer-rise fault models, estimated initial sea surface displacement, and calculated tsunami propagation. The fault models were based on high-resolution bathymetric surveys, as well as active and passive seismic observations^44–51^. The survey results showed that the upper edges of outer-rise faults reach the seafloor, have dip angles of 45–75°, and that the thickness of the seismogenic zone for normal faulting is about 40 km. Using an earthquake scaling law^52^ and these survey results, Baba et al.^41^ proposed 33 possible outer-rise faults in the Japan Trench. Because these predictions include uncertainties, they also tested variations of the predictions resulting from uncertainties in the assumed parameters. This study expands on the results of Baba et al.^41^, which assumed a rigidity of 50 GPa, which is too small considering S-wave velocity ($V_{s}$ ≈ 4.5 km/s) in the region. Based on the theory of elasticity, the rigidity can be calculated as $\rho V_{s}^{2}$, where $\rho$ is the density of the surrounding media. Therefore, in this study, we increased the rigidity to 65 GPa assuming an average density of $\rho$ ≈ 3,200 kg/m^3^ by 40 km depth below the seafloor. The parameters for the 33 outer-rise faults used in the tsunami calculations are reported in Table S1. Whereas Baba et al.^41^ used a single computational grid, we applied a nested grid system (Fig. 2) to improve tsunami prediction accuracy.

One of the 33 proposed faults corresponds to the 1933 Showa-Sanriku fault. If our modeling procedures are appropriate, one of the tsunamis calculated from the corresponding fault should agree with the observed inundation heights of the 1933 tsunami compiled in the Japan tsunami trace database^53^. Therefore, we compare the observed and calculated tsunami inundation heights from all 33 fault models.

Importantly, during the tsunami calculations, we observed that the non-dispersive model does not always provide larger tsunami heights than the dispersive model. In dispersive calculations of the 1933 Showa-Sanriku outer-rise tsunami, offshore frequency dispersions were apparent, resulting in tsunami inundation heights that were 10% larger than those in the non-dispersive tsunami calculations. Moreover, the dispersive tsunami calculations better predicted observed inundation heights than the non-dispersive calculations. These results call for greater attention to the effects of dispersion when making the deterministic tsunami hazard maps.

## Tsunami calculation results

**Figure 3:**
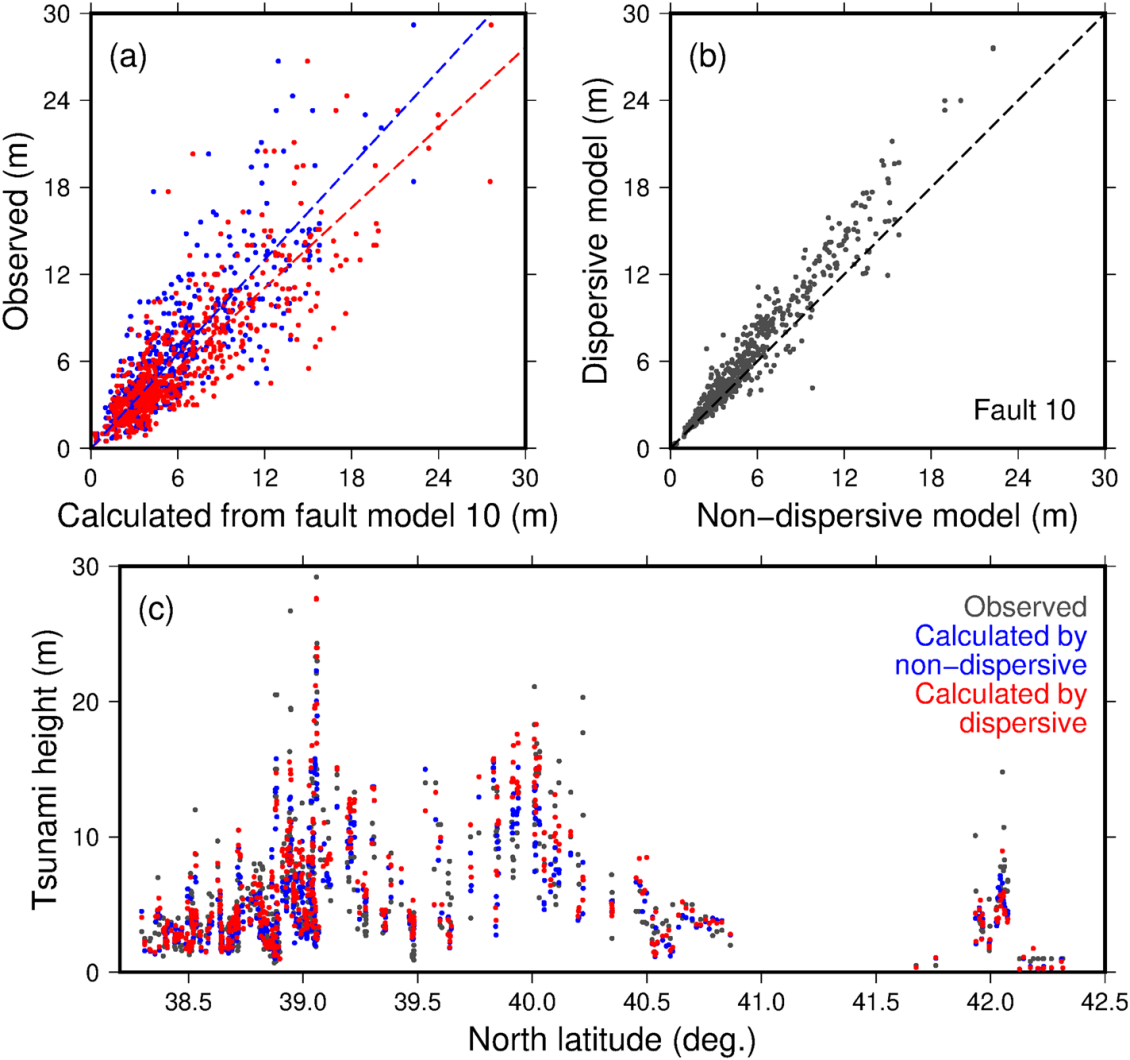
(**a**) Correlation between the observed inundation heights of the 1933 Showa-Sanriku tsunami and the calculated heights for fault model 10 using non-dispersive and dispersive equations (blue and red dots, respectively); the dashed lines indicate regression lines. (**b**) Correlation between tsunami inundation heights obtained from non-dispersive and dispersive calculations. The black dashed line indicates a 1:1 correspondence. (**c**) Distributed plots of observed and calculated inundation heights.

**Figure 4:**
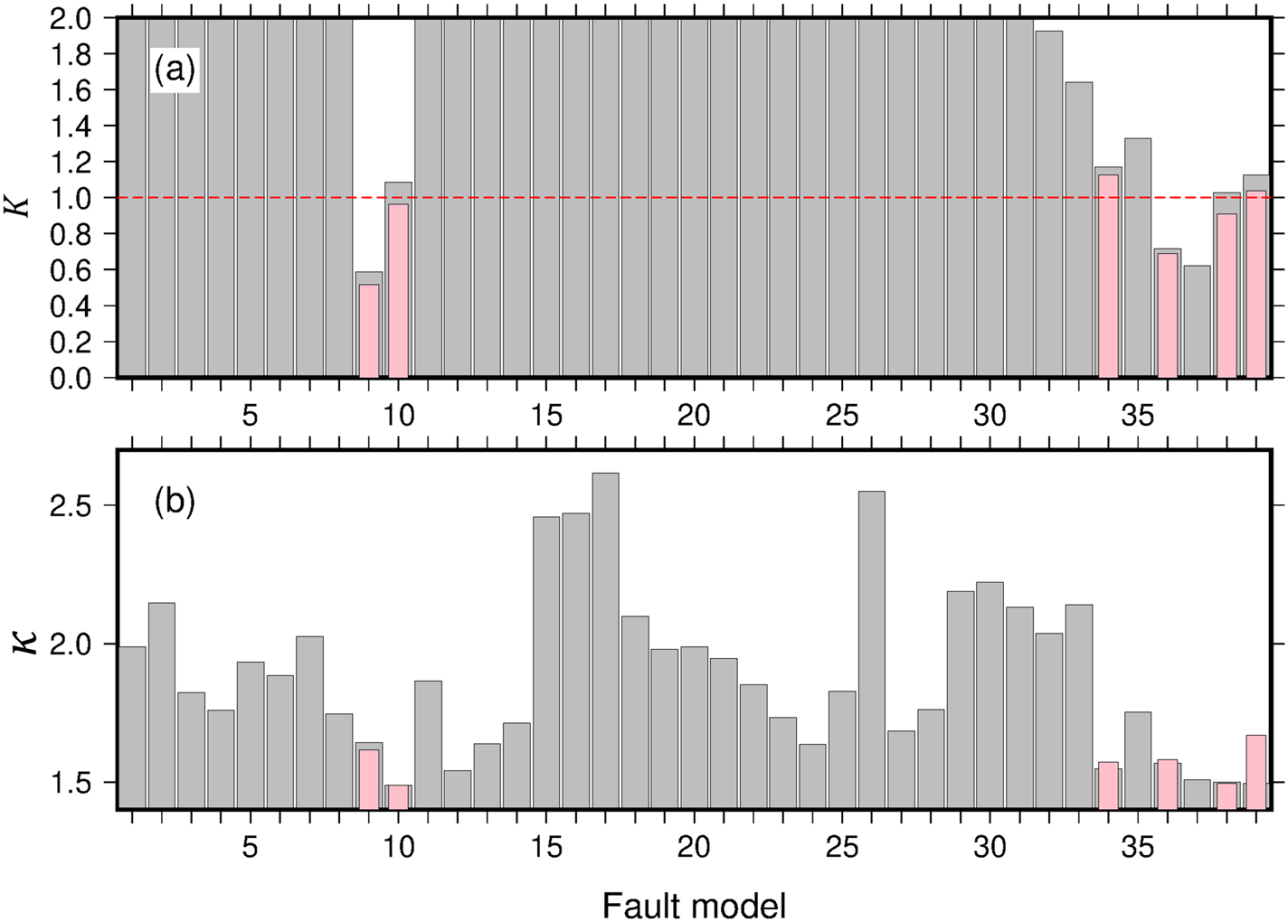
Histograms for (**a**) *K* and (**b**) *κ* for all fault models obtained with the non-dispersive and dispersive equations (gray and pink bars, respectively). The dashed red line indicates *K* = 1, representing a good match between the observations and predictions. The dispersive model was calculated only for fault models 9, 10, 34, 36, 38, and 39 because of computational resource limitations.

The outer-rise tsunamis were calculated from the 33 fault models by solving the nonlinear long-wave (non-dispersive) equations in a finite difference scheme. Fault model 10 (*L* = 218 km, *M*_*w*_ = 8.31, Table S1) best simulated the observed tsunami inundation heights of the 1933 tsunami with *K* = 1.09 and *κ* = 1.49 (Figs. 3 and 4), where *K* is the geometric average of the ratio between the observed and calculated tsunami inundation heights (values close to 1 mean well-predicted) and *κ* is the geometric standard deviation indicating the variance of *K*^54^. We applied the same procedure to evaluate several previously proposed fault models of the 1933 Showa-Sanriku earthquake^19–21, 55^ (models 34–38 in Table S1). Fault model 38^55^ was in good agreement with the observed inundation data (*K* = 1.03 and *κ* = 1.50). Figure 4 shows *K* and *κ* for all fault models presented in Table S1. Fault model 10 was comparable with the previously proposed fault models for the 1933 Showa-Sanriku earthquake. Whereas the previous studies^19, 21, 55^ adjusted the fault parameters to predict the observed 1933 tsunami, we implemented the tsunami calculations using a completely forward-looking method. We conclude that our tsunami prediction procedures, including the method to construct the fault models, are appropriate for predicting tsunamis caused by future outer-rise earthquakes in the Japan Trench.

We recalculated the outer-rise tsunamis from fault models 9, 10, 34, 36, and 38 (Table S1) using the dispersive equations. In the dispersive tsunami modeling, the tsunami caused by fault model 10 showed the best match with the observed inundation heights of the 1933 tsunami (Figs. 3 and 4; *K* = 0.97 and *κ* = 1.49). The decrease in *K* from 1.09 to 0.97 indicates that the tsunami inundation heights in the dispersive calculations were about 10% larger than those in the non-dispersive calculations, resulting in better agreement with the observed data.

## Discussion

Surprisingly, the dispersive calculations amplified the tsunami inundation heights in the coastal region by about 10%. This amplification is a result of the characteristic shape of the initial sea surface displacement by outer-rise earthquakes. Outer-rise earthquakes on high-angle normal faults cause a narrow band of subsidence as the initial tsunamigenic deformation. Figure 1b and Movie S1b show tsunami propagations of a downwardly convex solitary wave obtained by the dispersive and non-dispersive calculations. Dispersive deformation of the downwardly convex solitary wave is upside-down relative to that of an upwardly convex solitary wave. Whereas dispersion reduces the maximum height of the upwardly convex solitary wave (Fig. 1a), it increases the maximum height of the downwardly convex solitary wave (Fig. 1b).

The difference in phase velocity between the initial and later parts of the tsunami wave causes shoaling, which increases the height of the water wave that propagates over the region from the deep ocean to the coast (Movie S2). The first part of the wave slows in shallow water, but the later part propagates faster over deep water, compressing the wave and making it higher. The shoaling effect is significant in the non-dispersive equations because the water depth solely controls the phase velocity. In contrast, in the dispersive equations, phase velocity is expressed as a function of water depth and wavelength. A short-wavelength wave in the dispersive equations propagates slower than one in the non-dispersive equations, which results in a gentle shoaling-induced increase in amplitude. In dispersive outer-rise tsunami calculations, a pulling-dominant, short-wavelength wave has the property of increasing height caused by dispersion, but the shoaling increase is smaller than that in non-dispersive calculations. The superposition of these two effects deforms the tsunami waveforms offshore. In an actual bathymetric setting, because we also need to consider tsunami focusing or defocusing caused by complicated bathymetry, numerical calculations need to include bathymetric, shoaling, and dispersive effects to accurately predict the outer-rise tsunamis.

**Figure 5:**
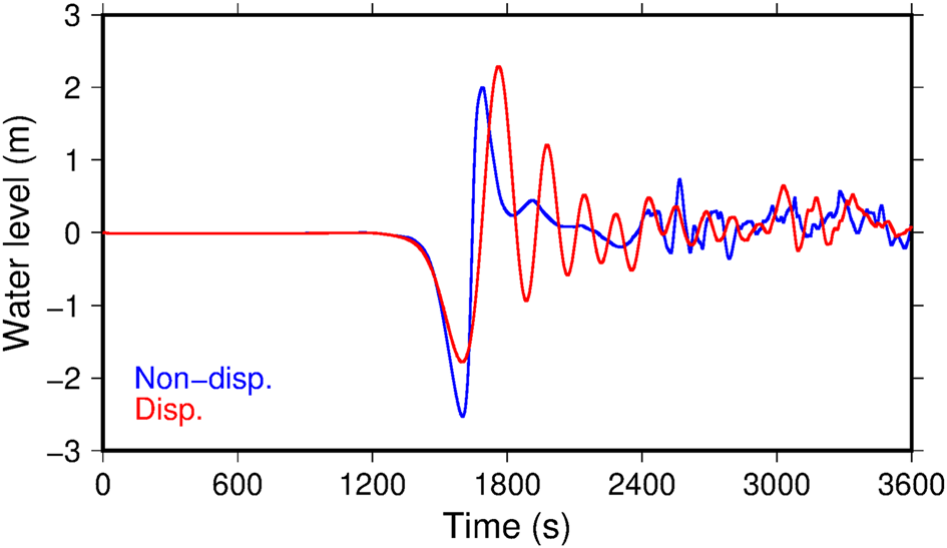
Calculated tsunami waveforms using the non-dispersive (blue) and dispersive (red) equations at a point 20 km offshore and in 200 m water depth (red circle in Fig. 2).

To evaluate dispersive deformation of the outer-rise tsunamis, in Fig. 5 we compare tsunami waveforms at a point where the water depth is 200 m and 20 km off the coast calculated using the dispersive and non-dispersive equations for fault model 10, i.e., where the amplitudes and wavelengths differ. We used the offshore tsunami waveform obtained from the dispersive equations as an incident wave and solved the tsunami propagation of the last 20 km using the non-dispersive equations. The tsunami inundation height in this test was the same as that when the dispersive equations were solved for all the regions. Therefore, in our current calculations, near-field dispersion in the 20 km closest to shore does not contribute to amplifying tsunami inundation heights. We conclude that offshore frequency dispersion amplified the tsunami inundation heights by about 10% during the 1933 Showa-Sanriku tsunami.

**Figure 6:**
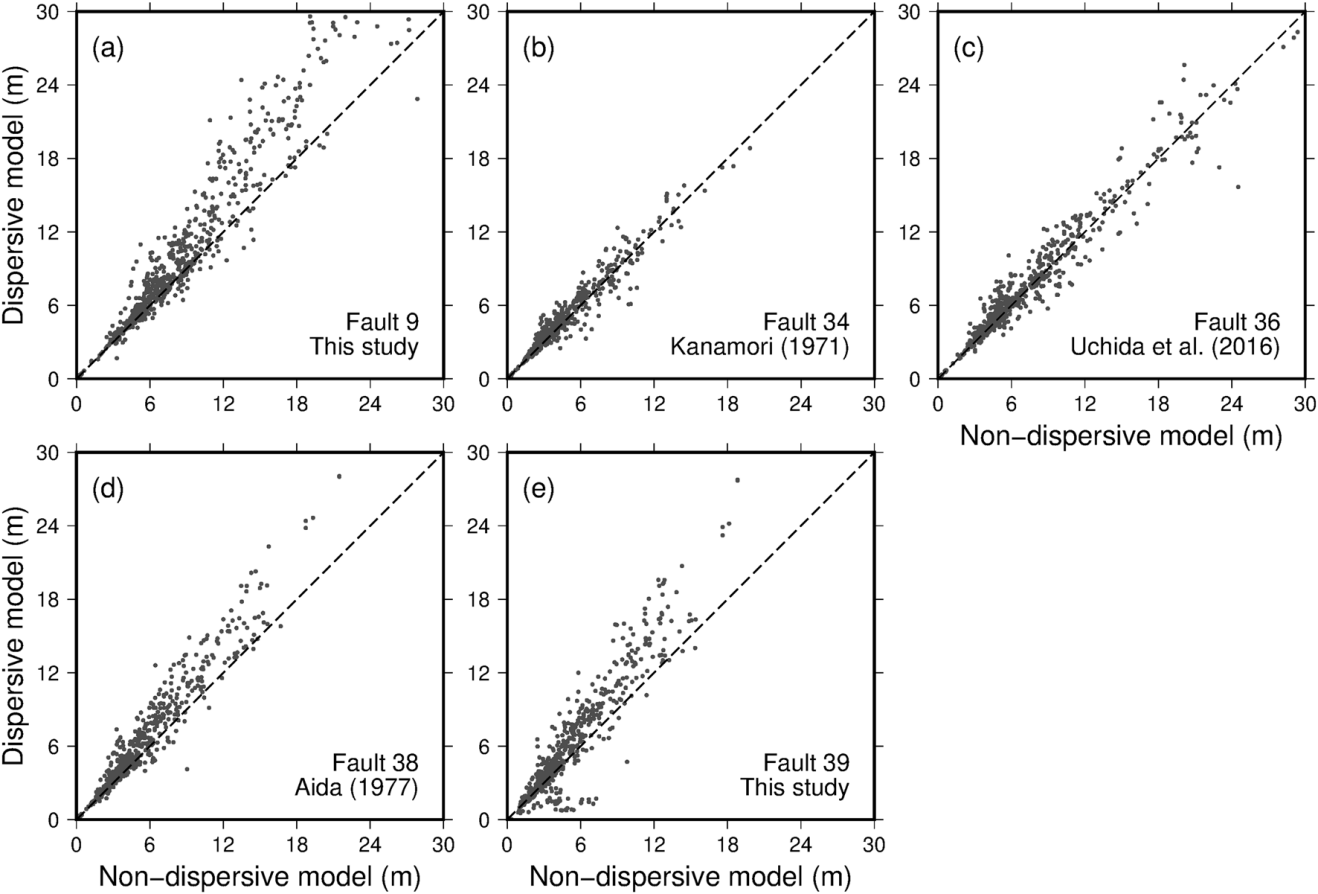
Correlation between tsunami inundation heights obtained from the non-dispersive and dispersive calculations. Fault models (**a**) 9 and (**e**) 39 are proposed in this study. Fault models (**b**) 34, (**c**) 36 and (**d**) 38 are based on Kanamori^20^, Uchida et al.^21^ and Aida^55^, respectively.

Similar to the plot shown in Fig. 3b for fault model 10, Fig. 6 compares tsunami inundation heights obtained using the non-dispersive and dispersive calculations for fault models 9, 34, 36, 38, and 39. Unfortunately, we were able to conduct dispersive wave calculations only on these faults because of computational resource limitations. The comparison points are the same as the on-land data points for the 1933 Showa-Sanriku tsunami (orange circles in Fig. 2). Tsunami inundation heights were not amplified due to dispersion for fault models 34^20^ and 36^21^ (Fig. 6b, c, respectively) because the fault parameters are inappropriate (e.g., the width is too long and the depth is too deep), resulting in tsunamis with longer wavelengths and less dispersivity. When the fault models proposed in this study are used (9 in Fig. 6a, 10 in Fig. 3b, and 39 in Fig. 6e), which are based on the reliable marine surveys, the dispersive wave model systematically predicted higher tsunami inundation heights than the non-dispersive model. Although nonlinearity has significant effects on tsunami-wave deformation very close to the coast, we can still confirm the dispersive amplification of tsunami inundation heights.

The question arises of whether non-dispersive equations underestimate tsunamis from interplate earthquakes. Therefore, we performed the same analysis for an ideal interplate earthquake (*M* 8.7, *L* = 200 km, *W* = 100 km, *D* = 10 m, dip = 10°) to evaluate the difference between the non-dispersive and dispersive models. The results show that the tsunami inundation heights of the two models are almost the same at the observed points of the 1933 tsunami (errors are about 0.5%). The wavelength of tsunamis generated by the interplate earthquakes is within the valid range of the long-wave approximation, so we can ignore the effect of tsunami dispersion for the great interplate earthquakes when making tsunami hazard maps. However, for tsunamis that travel across the ocean, the effect of dispersion becomes important over their long propagation distances^56^.

**Figure 7:**
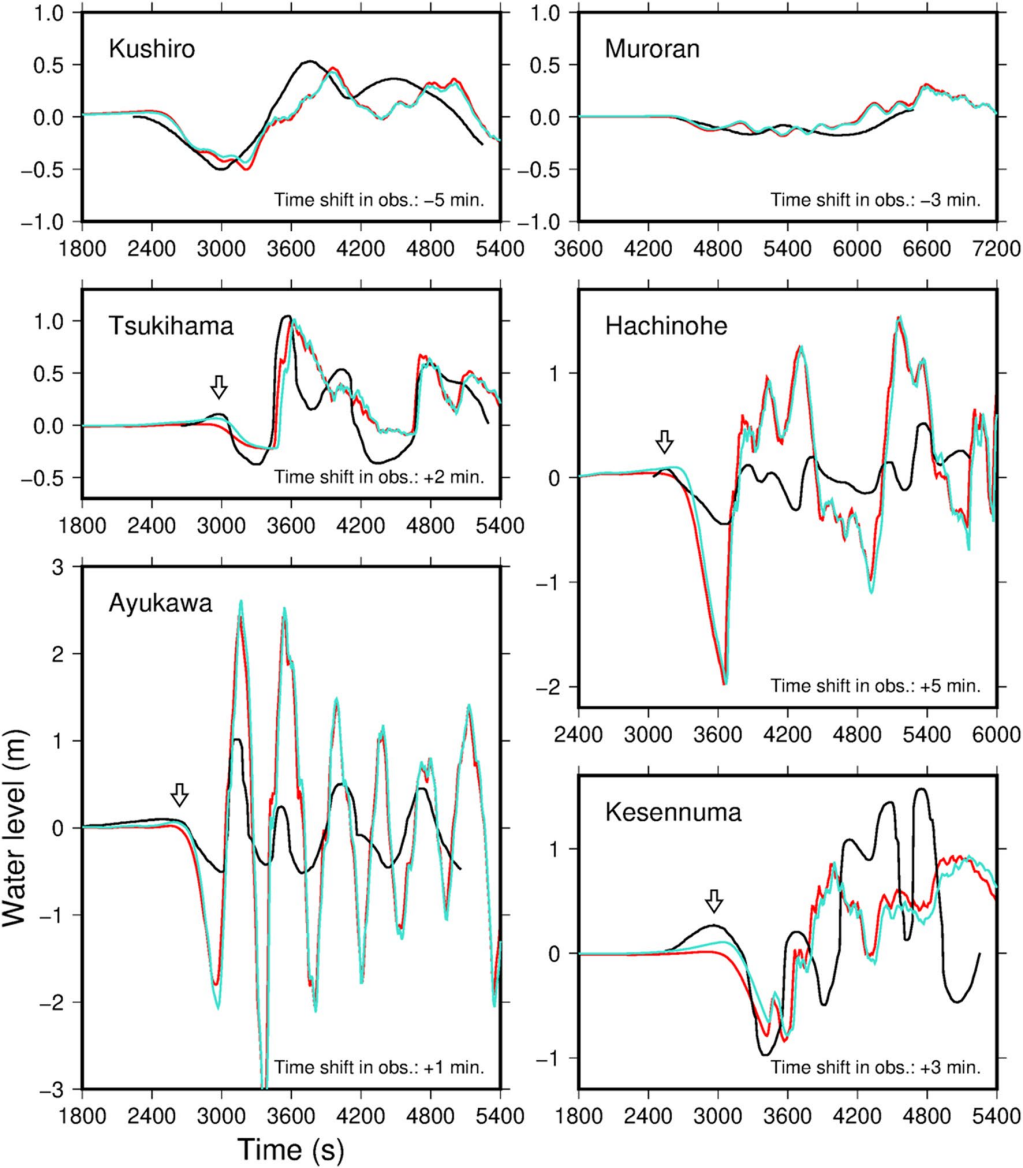
Observed and calculated tsunami waveforms at the tide gauges (see Fig. 2 and Table S2 for locations). The black waveforms are observed tsunamis, time-shifted to match the computed tsunami arrivals. The arrows show the first rise of the observed tsunami waveforms. The red and cyan waveforms are the respective computed tsunamis from fault models 10 and 39 (Table S1) using the dispersive equations. Time = 0 s is the earthquake origin time.

Several tide gauges along the coast recorded the 1933 Showa-Sanriku tsunami^19^. Figure 7 compares the recorded tsunami waveforms to those computed with fault model 10 using the dispersive equations. Because the historical paper records were of low quality, we shifted the observed tsunami waveforms by up to 5 min to match the computed tsunami arrivals shown in Fig. 7. The agreement between the computed and observed tsunami waveforms is relatively good at the Kushiro, Muroran, Tsukihama, and Kesennuma stations, although the coastal topography used in the tsunami calculations is different from that in 1933. At Hachinone, the calculated and recorded tsunami amplitudes differ. This difference may stem from the incompleteness of the bathymetric data. There is a discrepancy in amplitude between the computed and observed waveforms at the Ayukawa station. The amplitude of the computed tsunami waveform is about three times larger than the observed amplitude. However, because the computed amplitude is consistent with neighboring observed tsunami inundation heights in the tsunami trace database, we assume that the tide gauge response at Ayukawa was insensitive and unable to observe the short-wavelength tsunami correctly.

The tsunami waveforms computed from fault model 10 did not produce the first small rise in water level that was observed at Hachinohe, Tsukihama, Kesennuma, and Ayukawa (arrows in Fig. 7). On the other hand, fault model 38^55^ (Table S1) successfully simulated this small rise (Fig. S1). Fault parameters of fault models 10 and 38 are similar except for the fault dip angle (60° and 45°, respectively). The gentle dip angle of fault model 38 produced the first small rise. However, the seismic imaging results show that the outer-rise faults are dipping steeply by about 75° at shallow depths, a well-constrained result given the high quality of the active-source surveys^44^. At greater depths, fault dip angles are unclear in the marine seismic surveys. Together, these results can be interpreted as curved or listric faults, with steeply dipping near the surface and leveling off with depth. Therefore, we made a new fault model that consisted of an upper half with a 75° dip and a lower half with a 45° dip (fault model 39, Table S1). The tsunami waveforms calculated using this fault model include the first small rise in water level (cyan waveforms in Fig. 7) and reproduce the tsunami inundation heights in the tsunami trace database with *K* = 1.04 and *κ* = 1.67 (Fig. 4).

## Concluding remarks

In this study, we analyzed the tsunami caused by the 1933 Showa-Sanriku outer-rise earthquake in detail using a forward-looking method. We conclude that pulling-dominant, short-wavelength tsunamis caused by outer-rise earthquakes are characteristically amplified by offshore frequency dispersion. This conclusion has significant implications because underestimations in deterministic tsunami hazard maps are directly related to tsunami damages. It was long believed that using non-dispersive equations avoided underestimating tsunami heights in all case, albeit with lower accuracy than predictions using dispersive equations. However, in the case of outer-rise tsunamis, non-dispersive equations may underestimate tsunami heights, and dispersive equations must be used for tsunami predictions. Although dispersive calculations are computationally intense, recent computational developments will soon make them more accessible.

In recent years, probabilistic tsunami hazard assessments have rapidly developed^57, 58^; they consider both unspecified and specific tsunami sources with uncertainties and provide the probability that a maximum tsunami height will exceed a certain height within a given time frame. Deterministic and probabilistic maps express tsunami hazards differently, and neither is superior; deterministic maps allow quick and appropriate evacuations whereas probabilistic maps with cost-benefit analysis guide the construction of seawalls and other infrastructure. Based on the tsunami calculations in this study, the difference between dispersive and non-dispersive tsunami predictions should therefore be incorporated as one of the uncertainty elements in probabilistic tsunami hazard assessments.

## Tsunami calculation method

We performed tsunami calculations using the following procedure. Vertical crustal displacement at the seafloor was calculated assuming that the crust is a homogeneous elastic half-space^59^, and horizontal movement effects were included^60^. A hydraulic filter based on linear potential theory^38, 61^ was applied to estimate the initial sea-surface displacement, which assumed a rise time of 30 s. To model tsunami propagation, we used nonlinear long-wave (non-dispersive) equations^36^ and nonlinear dispersive equations^37, 38^. The time integrations were solved using JAGURS^62^, open-source software that solves either the non-dispersive or dispersive equations with a nested algorithm using a leapfrog, staggered-grid, finite-difference method.

For bathymetry, we used the Global tsunami Terrain Model^63^ (GtTM), which compiles open-source data around Japan to make 2 arc-sec gridded data. We resampled the GtTM to make a nested grid system consisting of 18 arc-sec, 6 arc-sec, and 2 arc-sec grids (Fig. 2). Although GtTM includes current data, it does not include human-made structures such as seawalls in the 2 arc-sec (~ 60 m) resolution grid, so it is more suitable for reproducing historical tsunamis such as the 1933 Showa-Sanriku tsunami than existing high-precision and high-resolution bathymetric datasets. The time-step width was set to 0.1 s to satisfy the stability conditions of the finite-difference calculation. The integration time was 150 min after the origin time of the 1933 tsunami, which included the maximum tsunami arrivals along the entire coast. We applied Manning’s law for the bottom friction with a roughness coefficient of 0.025 s/m^1/3^ and an absorbing condition at the outer boundary of the computational region.

We compared the calculated and observed tsunami heights from the 1933 Showa-Sanriku tsunami in the Japan tsunami trace database compiled by Tohoku University and the Nuclear Regulation Authority^53^. We used only the observed tsunami data considered to be most reliable (category A) in our comparisons. The total number of points to compare was 670 (Fig. 2). If the computed tsunami did not reach the observed point on the slope, the tsunami height at the nearest computation point was used for the comparison. Using the database, we calculated Aida’s^54^ *K* and *κ* for all of the calculated tsunamis in this study to find the best-fit fault model for the 1933 Showa-Sanriku earthquake. We also compared the calculated and observed tsunami waveforms at the tide gauge stations (Fig. 2). The observed tsunami waveforms were digitized using figures in Abe^19^. If the tide gauges were on land in our computational bathymetric data, we shifted the tide gauge location to the nearest sea point (Table S2).

## Supplementary Information

Supplementary Information 1.

Supplementary Information 2.

Supplementary Information 3.

## Data Availability

We used tsunami software JAGURS^62^ provided in an online repository at https://doi.org/10.5281/zenodo.3737816, and bathymetric data GtTM^63^ at doi: https://doi.org/10.17598/NIED.0021. The Japan tsunami trace database^53^ is at https://tsunami-db.irides.tohoku.ac.jp/tsunami/?LANG=-2.
