# Supplementary material for: Frequency dispersion amplifies tsunamis caused by outer-rise normal faults
Source: Sci Rep. 2021 Oct 8;11:20064. doi: 10.1038/s41598-021-99536-x (PMC8501110; doi:10.1038/s41598-021-99536-x)
Supplement: Supplementary file 1 — Supplementary Information 1. [file 41598_2021_99536_MOESM1_ESM.docx]

**Supplementary Information for**

**Frequency Dispersion Amplifies Tsunamis Caused by Outer-rise Normal Faults**

**Baba, T. et al., Scientific Reports**

This document contains supplementary one figure and two tables. Figure S1 compares observed and calculated tsunami waveforms at tide gauges. Tables S1 shows outer-rise fault parameters used in this study. Table S2 is location of the tide gauges.

**Figure, table and movie captions**

**Figure S1.** Observed and calculated tsunami waveforms at the tide gauges (see Fig. 2 and Table S2 for locations). The black waveforms are observed tsunamis, but the time is shifted to match the computed tsunami arrivals. Computed tsunami waveforms are from fault models 34 (red), 36 (purple), and 38 (green) using the dispersive equations.

**Table S1.** Outer-rise fault parameters assuming a rigidity of 65 GPa.

**Table S2.** Location of the tide gauges

**Movie S1.** Non-dispersive (blue) and dispersive (red) propagation of Gaussian soliton waves. (a) A positive (upwardly convex) incident wave. (b) A negative (downwardly convex) incident wave. The waves propagate in the *x*-direction over a water depth of 4,000 m. Dispersion creates wave trains and increases the wave height in (b).

**Movie S2.** Non-dispersive (blue) and dispersive (red) propagation of a tsunami caused by an outer-rise fault along the white line in the map on the left. Water depth is shown by the black line. The tsunami is deformed by shoaling and dispersion.


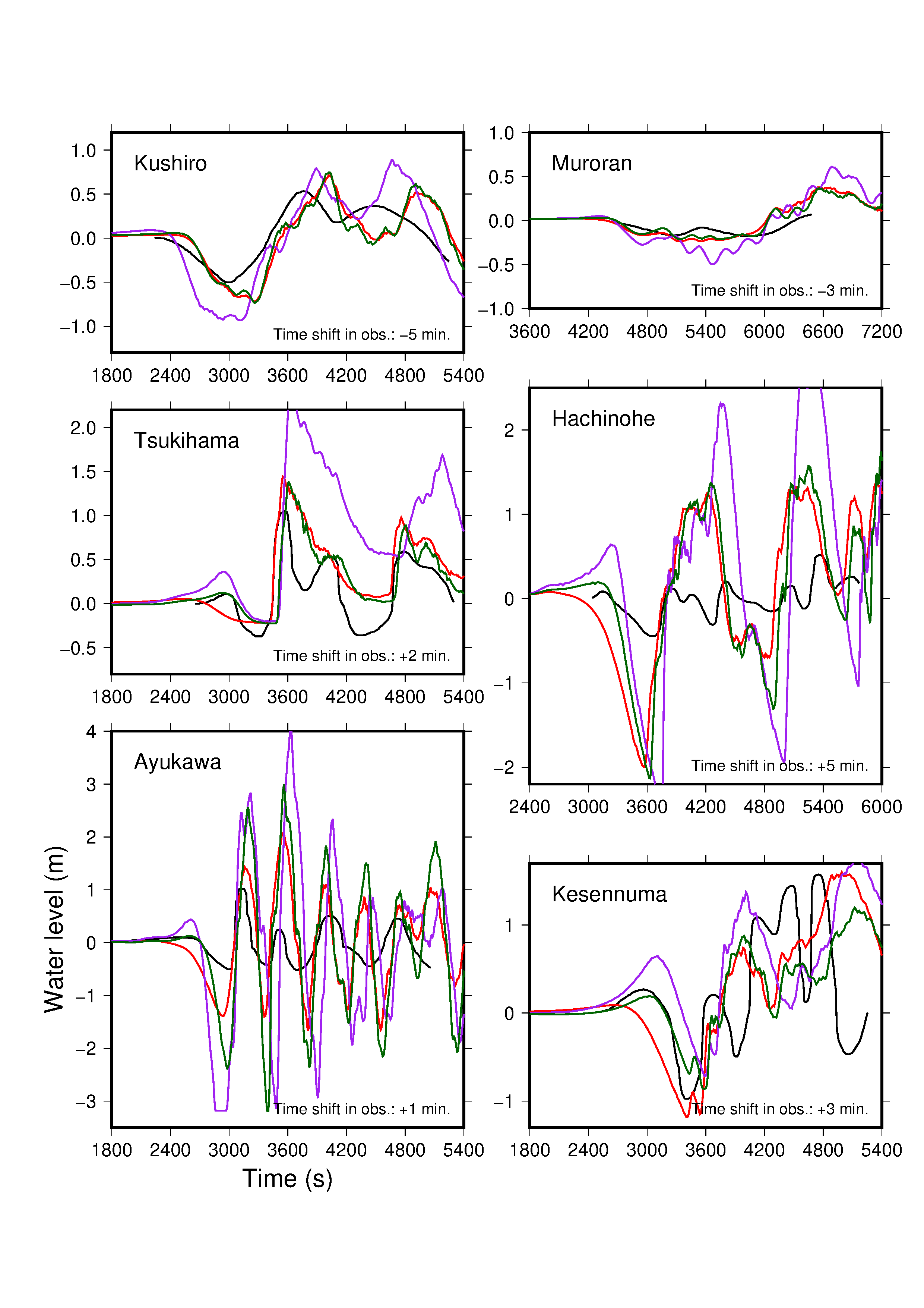


**Figure S1.** Observed and calculated tsunami waveforms at the tide gauges (see Fig. 2 and Table S2 for locations). The black waveforms are observed tsunamis, but the time is shifted to match the computed tsunami arrivals. Computed tsunami waveforms are from fault models 34 (red), 36 (purple), and 38 (green) using the dispersive equations.

**Table S1.** Outer-rise fault parameters assuming a rigidity of 65 GPa

| **ID** | **Lat.**  **(°)^a^** | **Long.**  **(°)^a^** | **Depth (km)** | ***L***  **(km)** | ***W***  **(km)** | **Dip (°)** | **Strike (°)** | **Rake (°)** | **Slip**  **(m)** | ***M*_w_** | **Reference** |
| --- | --- | --- | --- | --- | --- | --- | --- | --- | --- | --- | --- |
| 1 | 38.1708 | 144.0276 | 0.1 | 46 | 46 | 60 | 180 | 270 | 0.32 | 7.03 | This study (modified from Baba et al., 2020) |
| 2 | 38.0249 | 144.1599 | 0.1 | 61 | 46 | 60 | 181 | 270 | 0.54 | 7.26 |  |
| 3 | 38.0646 | 144.2033 | 0.1 | 45 | 45 | 60 | 152 | 270 | 0.32 | 7.02 |  |
| 4 | 38.9365 | 144.4128 | 0.1 | 42 | 42 | 60 | 190 | 270 | 0.30 | 6.95 |  |
| 5 | 39.0975 | 144.2551 | 0.1 | 65 | 46 | 60 | 190 | 270 | 0.61 | 7.32 |  |
| 6 | 39.5916 | 144.2561 | 0.1 | 114 | 46 | 60 | 177 | 270 | 1.71 | 7.78 |  |
| 7 | 39.5916 | 144.2561 | 0.1 | 106 | 46 | 60 | 168 | 270 | 1.48 | 7.71 |  |
| 8 | 40.0254 | 144.5111 | 0.1 | 76 | 46 | 60 | 180 | 270 | 0.81 | 7.45 |  |
| 9 | 40.2224 | 144.8678 | 0.1 | 332 | 46 | 60 | 181 | 270 | 12.13 | 8.66 |  |
| 10 | 40.2224 | 144.8678 | 0.1 | 218 | 46 | 60 | 189 | 270 | 5.62 | 8.31 |  |
| 11 | 40.3472 | 145.1102 | 0.1 | 61 | 46 | 60 | 185 | 270 | 0.55 | 7.27 |  |
| 12 | 39.8068 | 144.8759 | 0.1 | 133 | 46 | 60 | 187 | 270 | 2.27 | 7.91 |  |
| 13 | 39.3941 | 144.9400 | 0.1 | 74 | 46 | 60 | 190 | 270 | 0.77 | 7.42 |  |
| 14 | 39.5788 | 144.3744 | 0.1 | 51 | 46 | 60 | 176 | 270 | 0.39 | 7.29 |  |
| 15 | 38.7381 | 144.9041 | 0.1 | 167 | 46 | 60 | 184 | 270 | 3.44 | 8.09 |  |
| 16 | 38.6148 | 144.2442 | 0.1 | 103 | 46 | 60 | 170 | 270 | 1.42 | 7.69 |  |
| 17 | 38.1362 | 144.0142 | 0.1 | 127 | 46 | 60 | 199 | 270 | 2.09 | 7.87 |  |
| 18 | 37.2671 | 143.8665 | 0.1 | 58 | 46 | 60 | 206 | 270 | 0.49 | 7.22 |  |
| 19 | 37.2784 | 144.5308 | 0.1 | 52 | 46 | 60 | 234 | 270 | 0.41 | 7.14 |  |
| 20 | 38.0318 | 144.1224 | 0.1 | 55 | 46 | 60 | 4 | 270 | 0.45 | 7.18 |  |
| 21 | 38.2164 | 144.2062 | 0.1 | 55 | 46 | 60 | 346 | 270 | 0.45 | 7.18 |  |
| 22 | 39.2673 | 144.3149 | 0.1 | 84 | 46 | 60 | 5 | 270 | 0.97 | 7.53 |  |
| 23 | 39.9867 | 144.3634 | 0.1 | 69 | 46 | 60 | 2 | 270 | 0.67 | 7.36 |  |
| 24 | 40.0149 | 144.6868 | 0.1 | 128 | 46 | 60 | 7 | 270 | 2.10 | 7.87 |  |
| 25 | 40.2359 | 144.5756 | 0.1 | 73 | 46 | 60 | 13 | 270 | 0.76 | 7.42 |  |
| 26 | 37.4764 | 143.9826 | 0.1 | 102 | 46 | 60 | 30 | 270 | 1.40 | 7.69 |  |
| 27 | 39.4220 | 144.8967 | 0.1 | 76 | 46 | 60 | 9 | 270 | 0.81 | 7.44 |  |
| 28 | 38.9404 | 144.6059 | 0.1 | 44 | 43 | 60 | 17 | 270 | 0.31 | 6.99 |  |
| 29 | 38.3397 | 144.5048 | 0.1 | 67 | 46 | 60 | 3 | 270 | 0.64 | 7.34 |  |
| 30 | 37.2620 | 144.4183 | 0.1 | 71 | 46 | 60 | 55 | 270 | 0.72 | 7.39 |  |
| 31 | 37.9659 | 144.9115 | 0.1 | 155 | 46 | 60 | 41 | 270 | 3.00 | 8.03 |  |
| 32 | 37.9652 | 144.9127 | 0.1 | 166 | 46 | 60 | 49 | 270 | 3.42 | 8.09 |  |
| 33 | 37.9659 | 144.9124 | 0.1 | 195 | 46 | 60 | 42 | 270 | 4.58 | 8.22 |  |
| 34 | 40.1600 | 144.5000 | 0.0 | 185 | 100 | 45 | 180 | 270 | 3.30 | 8.33 | Kanamori (1971) |
| 35 | 44.0000 | 144.5000 | 0.0 | 270 | 70 | 30 | 180 | 270 | 3.30 | 8.34 | Abe (1978) |
| 36 | 40.3900 | 145.3100 | 4.0 | 278 | 58 | 60 | 188 | 270 | 4.50 | 8.58 | Uchida et al. (2016) |
|  | 40.4949 | 144.3974 | 4.0 | 278 | 58 | 60 | 8 | 270 | 4.50 |  |  |
| 37 | 40.3900 | 145.3100 | 4.0 | 278 | 58 | 60 | 188 | 270 | 9.00 | 8.58 |  |
| 38 | 40.1600 | 144.500 | 1.0 | 185 | 50 | 45 | 180 | 270 | 6.60 | 8.33 | Aida (1977) |
| 39 | 40.22234 | 144.8678 | 0.1 | 218 | 23 | 75 | 189 | 270 | 5.62 | 8.31 | This study |
|  | 40.23058 | 144.7982 | 22.4 | 218 | 23 | 45 | 189 | 270 | 5.62 |  |  |

^a^Latitude and longitude mark the northern end of the upper edge of the fault plane

**Table S2.** Location of the tide gauges

| **Station name** | **Lat.(°)** | **Long.(°)** |
| --- | --- | --- |
| Kushiro | 42.9761 | 144.3706 |
| Muroran | 42.3417 | 140.9567 |
| Hachinohe | 40.5289 | 141.5314 |
| Kesennnuma | 38.9041 | 141.5861 |
| Tsukihama | 38.5717 | 141.4500 |
| Ayukawa | 38.2936 | 141.5086 |
